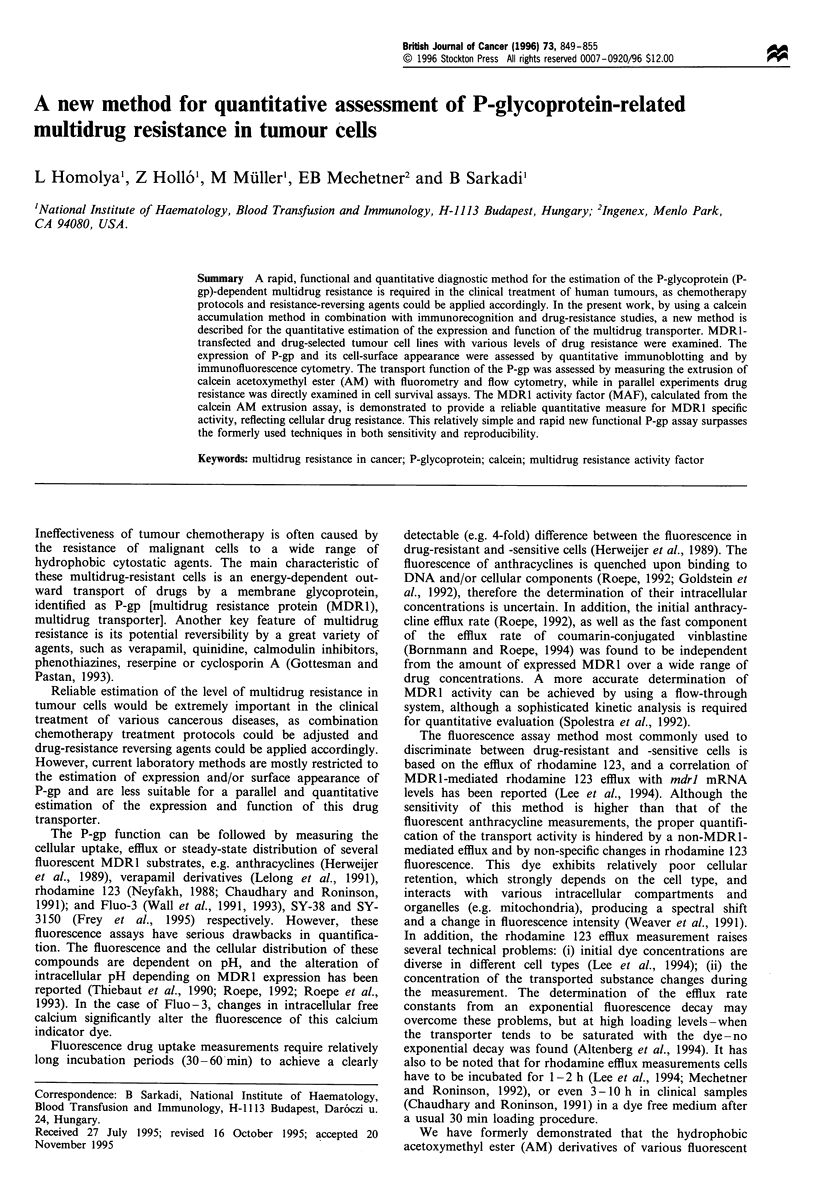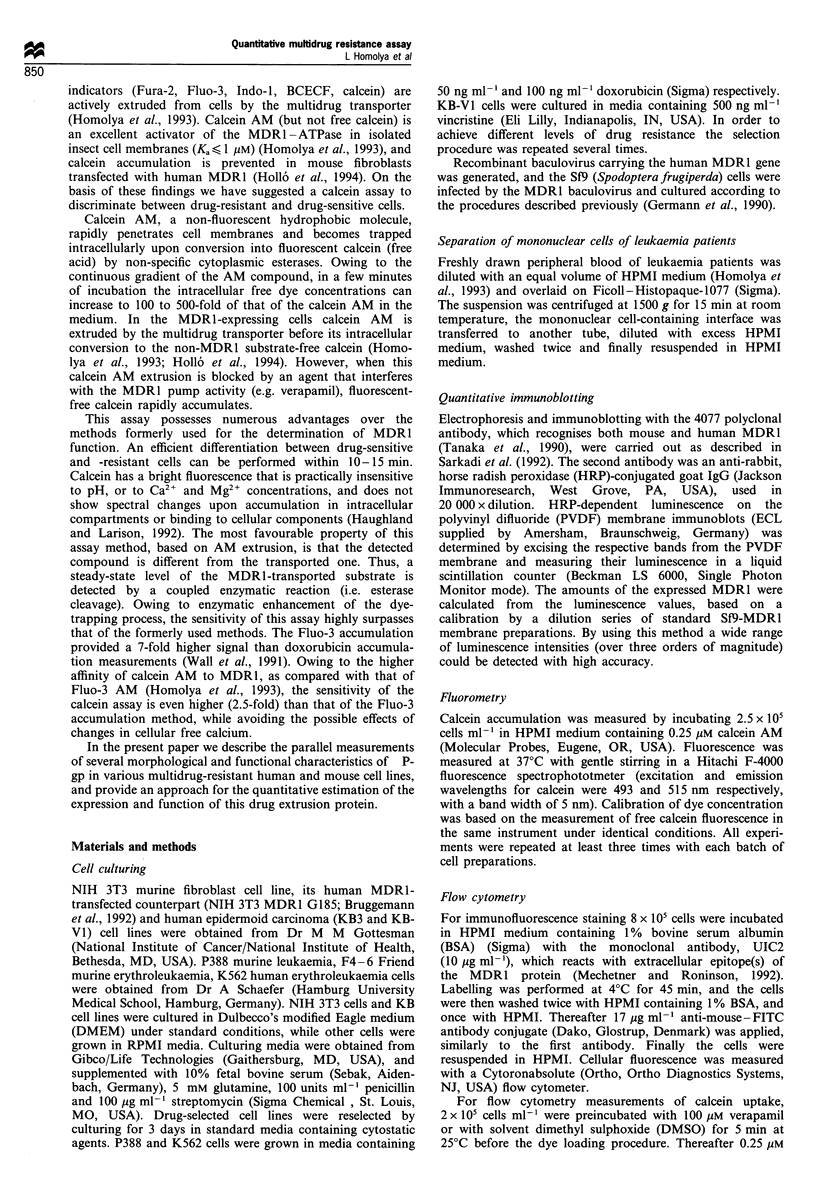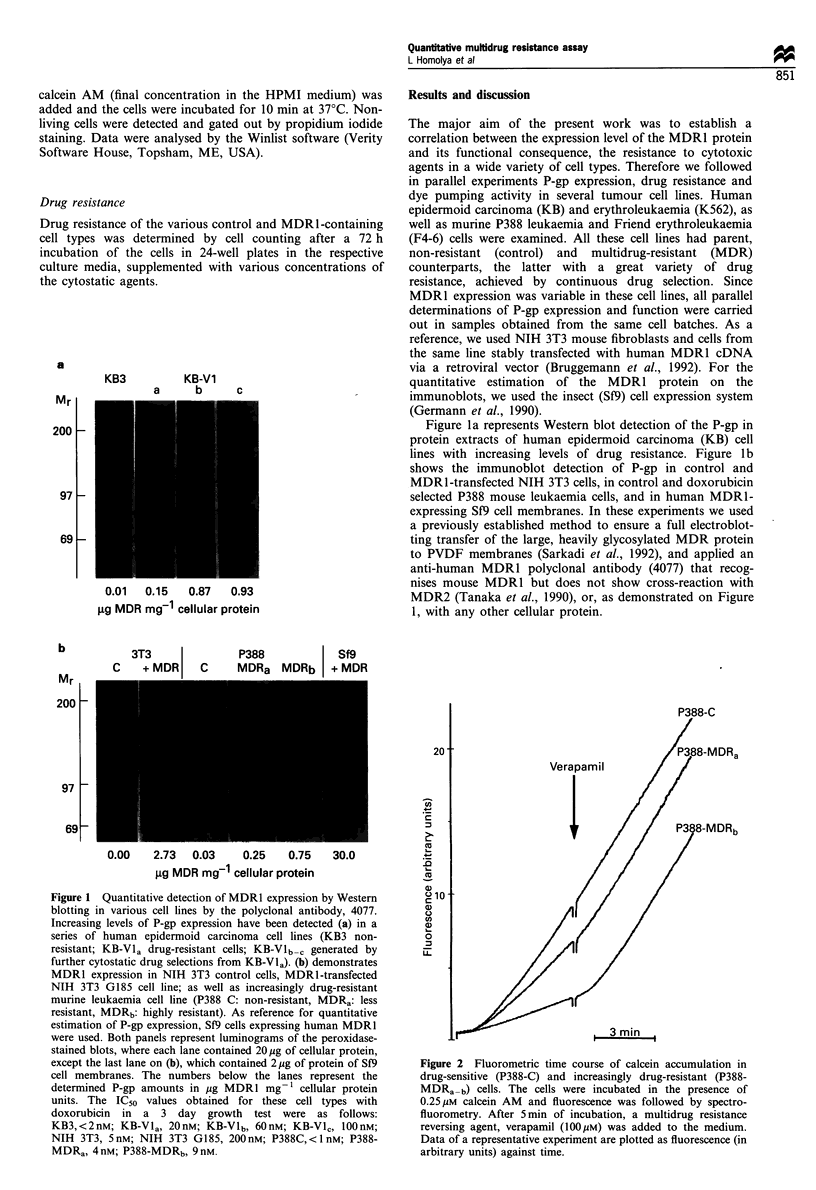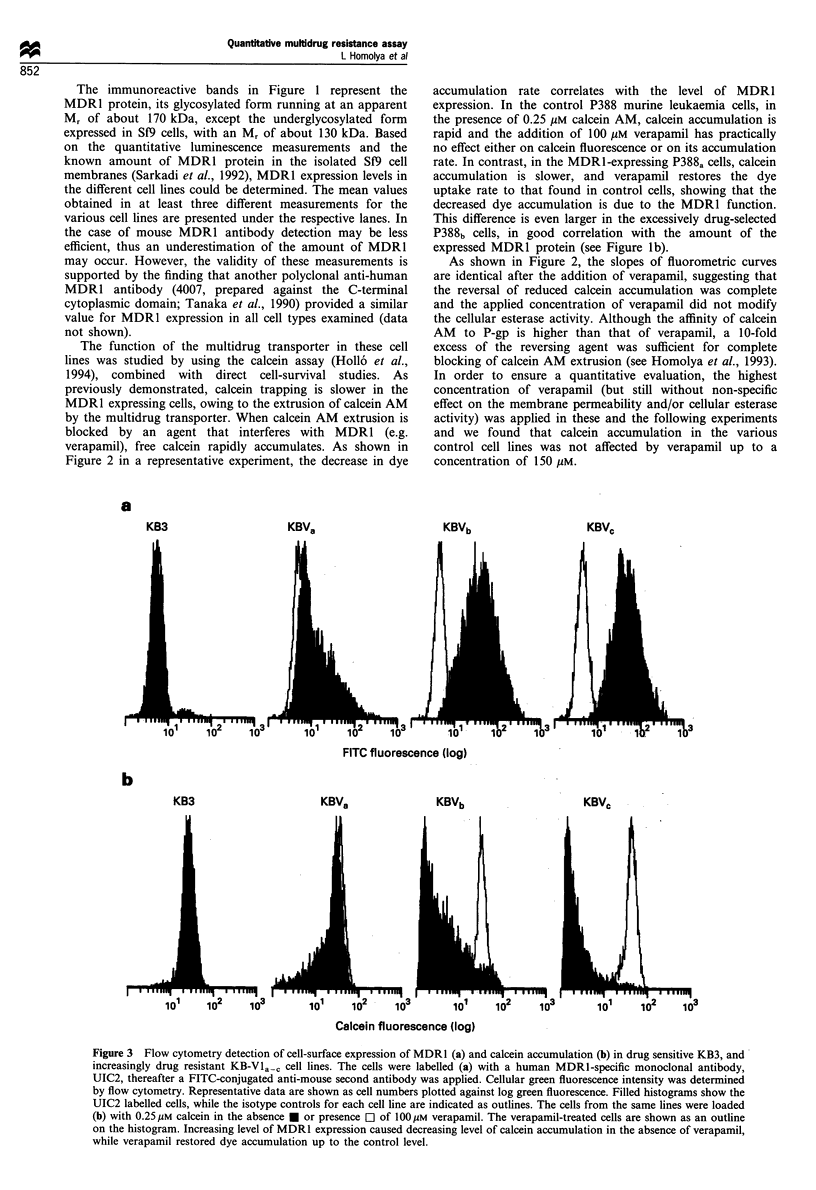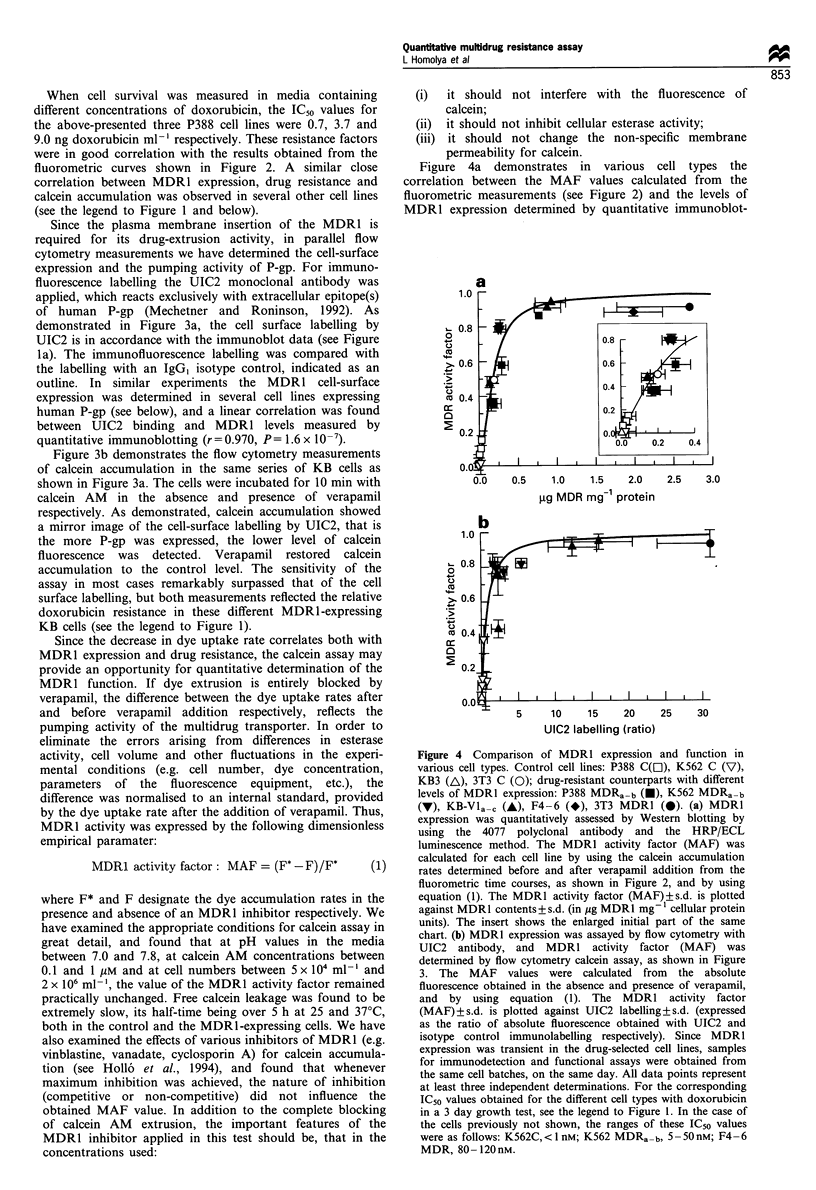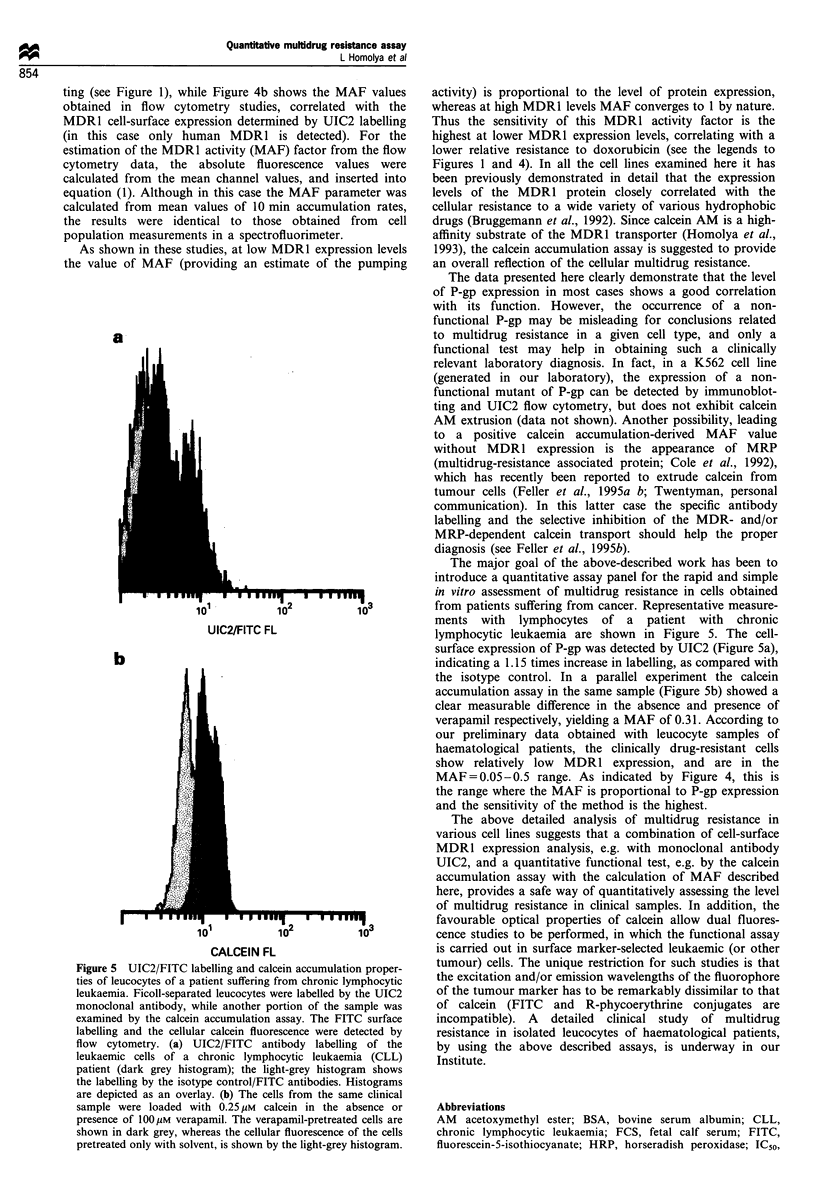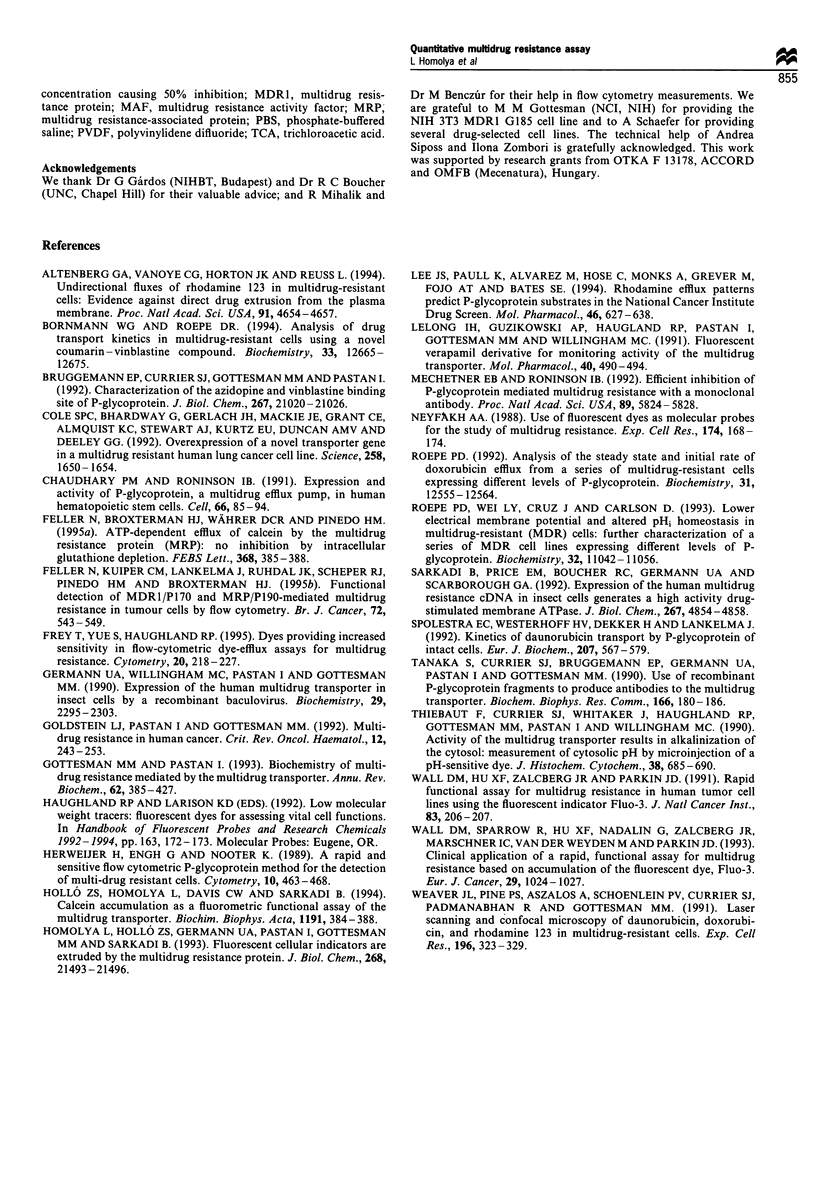# A new method for a quantitative assessment of P-glycoprotein-related multidrug resistance in tumour cells.

**DOI:** 10.1038/bjc.1996.151

**Published:** 1996-04

**Authors:** L. Homolya, M. Holló, M. Müller, E. B. Mechetner, B. Sarkadi

**Affiliations:** National Institute of Haematology, Blood Transfusion and Immunology, Budapest, Hungary.

## Abstract

**Images:**


British Journal of Cancer (1996) 73, 849-855
© 1996 Stockton Press All rights reserved 0007-0920/96 $12.00

### A new method for quantitative assessment of P-glycoprotein-related multidrug resistance in tumour cells

L Homolya[1], Z Holló[1], M Müller[1], EB Mechetner[2] and B Sarkadi[1]

[1]*National Institute of Haematology, Blood Transfusion and Immunology, H-1113 Budapest, Hungary;* [2]*Ingenex, Menlo Park, CA 94080, USA.*

**Summary** A rapid, functional and quantitative diagnostic method for the estimation of the P-glycoprotein (P-gp)-dependent multidrug resistance is required in the clinical treatment of human tumours, as chemotherapy protocols and resistance-reversing agents could be applied accordingly. In the present work, by using a calcein accumulation method in combination with immunorecognition and drug-resistance studies, a new method is described for the quantitative estimation of the expression and function of the multidrug transporter. MDR1-transfected and drug-selected tumour cell lines with various levels of drug resistance were examined. The expression of P-gp and its cell-surface appearance were assessed by quantitative immunoblotting and by immunofluorescence cytometry. The transport function of the P-gp was assessed by measuring the extrusion of calcein acetoxymethyl ester (AM) with fluorometry and flow cytometry, while in parallel experiments drug resistance was directly examined in cell survival assays. The MDR1 activity factor (MAF), calculated from the calcein AM extrusion assay, is demonstrated to provide a reliable quantitative measure for MDR1 specific activity, reflecting cellular drug resistance. This relatively simple and rapid new functional P-gp assay surpasses the formerly used techniques in both sensitivity and reproducibility.

**Keywords:** multidrug resistance in cancer; P-glycoprotein; calcein; multidrug resistance activity factor

Ineffectiveness of tumour chemotherapy is often caused by the resistance of malignant cells to a wide range of hydrophobic cytostatic agents. The main characteristic of these multidrug-resistant cells is an energy-dependent outward transport of drugs by a membrane glycoprotein, identified as P-gp [multidrug resistance protein (MDR1), multidrug transporter]. Another key feature of multidrug resistance is its potential reversibility by a great variety of agents, such as verapamil, quinidine, calmodulin inhibitors, phenothiazines, reserpine or cyclosporin A (Gottesman and Pastan, 1993).

Reliable estimation of the level of multidrug resistance in tumour cells would be extremely important in the clinical treatment of various cancerous diseases, as combination chemotherapy treatment protocols could be adjusted and drug-resistance reversing agents could be applied accordingly. However, current laboratory methods are mostly restricted to the estimation of expression and/or surface appearance of P-gp and are less suitable for a parallel and quantitative estimation of the expression and function of this drug transporter.

The P-gp function can be followed by measuring the cellular uptake, efflux or steady-state distribution of several fluorescent MDR1 substrates, e.g. anthracyclines (Herweijer *et al.*, 1989), verapamil derivatives (Lelong *et al.*, 1991), rhodamine 123 (Neyfakh, 1988; Chaudhary and Roninson, 1991); and Fluo-3 (Wall *et al.*, 1991, 1993), SY-38 and SY-3150 (Frey *et al.*, 1995) respectively. However, these fluorescence assays have serious drawbacks in quantification. The fluorescence and the cellular distribution of these compounds are dependent on pH, and the alteration of intracellular pH depending on MDR1 expression has been reported (Thiebaut *et al.*, 1990; Roepe, 1992; Roepe *et al.*, 1993). In the case of Fluo-3, changes in intracellular free calcium significantly alter the fluorescence of this calcium indicator dye.

Fluorescence drug uptake measurements require relatively long incubation periods (30-60 min) to achieve a clearly detectable (e.g. 4-fold) difference between the fluorescence in drug-resistant and -sensitive cells (Herweijer *et al.*, 1989). The fluorescence of anthracyclines is quenched upon binding to DNA and/or cellular components (Roepe, 1992; Goldstein *et al.*, 1992), therefore the determination of their intracellular concentrations is uncertain. In addition, the initial anthracycline efflux rate (Roepe, 1992), as well as the fast component of the efflux rate of coumarin-conjugated vinblastine (Bornmann and Roepe, 1994) was found to be independent from the amount of expressed MDR1 over a wide range of drug concentrations. A more accurate determination of MDR1 activity can be achieved by using a flow-through system, although a sophisticated kinetic analysis is required for quantitative evaluation (Spolestra *et al.*, 1992).

The fluorescence assay method most commonly used to discriminate between drug-resistant and -sensitive cells is based on the efflux of rhodamine 123, and a correlation of MDR1-mediated rhodamine 123 efflux with *mdr1* mRNA levels has been reported (Lee *et al.*, 1994). Although the sensitivity of this method is higher than that of the fluorescent anthracycline measurements, the proper quantification of the transport activity is hindered by a non-MDR1-mediated efflux and by non-specific changes in rhodamine 123 fluorescence. This dye exhibits relatively poor cellular retention, which strongly depends on the cell type, and interacts with various intracellular compartments and organelles (e.g. mitochondria), producing a spectral shift and a change in fluorescence intensity (Weaver *et al.*, 1991). In addition, the rhodamine 123 efflux measurement raises several technical problems: (i) initial dye concentrations are diverse in different cell types (Lee *et al.*, 1994); (ii) the concentration of the transported substance changes during the measurement. The determination of the efflux rate constants from an exponential fluorescence decay may overcome these problems, but at high loading levels-when the transporter tends to be saturated with the dye-no exponential decay was found (Altenberg *et al.*, 1994). It has also to be noted that for rhodamine efflux measurements cells have to be incubated for 1-2 h (Lee *et al.*, 1994; Mechetner and Roninson, 1992), or even 3-10 h in clinical samples (Chaudhary and Roninson, 1991) in a dye free medium after a usual 30 min loading procedure.

We have formerly demonstrated that the hydrophobic acetoxymethyl ester (AM) derivatives of various fluorescent

Correspondence: B Sarkadi, National Institute of Haematology, Blood Transfusion and Immunology, H-1113 Budapest, Daróczi u. 24, Hungary.
Received 27 July 1995; revised 16 October 1995; accepted 20 November 1995

indicators (Fura-2, Fluo-3, Indo-1, BCECF, calcein) are actively extruded from cells by the multidrug transporter (Homolya *et al.*, 1993). Calcein AM (but not free calcein) is an excellent activator of the MDR1–ATPase in isolated insect cell membranes ($K_a \leq 1$ μM) (Homolya *et al.*, 1993), and calcein accumulation is prevented in mouse fibroblasts transfected with human MDR1 (Holló *et al.*, 1994). On the basis of these findings we have suggested a calcein assay to discriminate between drug-resistant and drug-sensitive cells.

Calcein AM, a non-fluorescent hydrophobic molecule, rapidly penetrates cell membranes and becomes trapped intracellularly upon conversion into fluorescent calcein (free acid) by non-specific cytoplasmic esterases. Owing to the continuous gradient of the AM compound, in a few minutes of incubation the intracellular free dye concentrations can increase to 100 to 500-fold of that of the calcein AM in the medium. In the MDR1-expressing cells calcein AM is extruded by the multidrug transporter before its intracellular conversion to the non-MDR1 substrate-free calcein (Homolya *et al.*, 1993; Holló *et al.*, 1994). However, when this calcein AM extrusion is blocked by an agent that interferes with the MDR1 pump activity (e.g. verapamil), fluorescent-free calcein rapidly accumulates.

This assay possesses numerous advantages over the methods formerly used for the determination of MDR1 function. An efficient differentiation between drug-sensitive and -resistant cells can be performed within 10–15 min. Calcein has a bright fluorescence that is practically insensitive to pH, or to $Ca^{2+}$ and $Mg^{2+}$ concentrations, and does not show spectral changes upon accumulation in intracellular compartments or binding to cellular components (Haughland and Larison, 1992). The most favourable property of this assay method, based on AM extrusion, is that the detected compound is different from the transported one. Thus, a steady-state level of the MDR1-transported substrate is detected by a coupled enzymatic reaction (i.e. esterase cleavage). Owing to enzymatic enhancement of the dye-trapping process, the sensitivity of this assay highly surpasses that of the formerly used methods. The Fluo-3 accumulation provided a 7-fold higher signal than doxorubicin accumulation measurements (Wall *et al.*, 1991). Owing to the higher affinity of calcein AM to MDR1, as compared with that of Fluo-3 AM (Homolya *et al.*, 1993), the sensitivity of the calcein assay is even higher (2.5-fold) than that of the Fluo-3 accumulation method, while avoiding the possible effects of changes in cellular free calcium.

In the present paper we describe the parallel measurements of several morphological and functional characteristics of P-gp in various multidrug-resistant human and mouse cell lines, and provide an approach for the quantitative estimation of the expression and function of this drug extrusion protein.

### Materials and methods

### *Cell culturing*

NIH 3T3 murine fibroblast cell line, its human MDR1-transfected counterpart (NIH 3T3 MDR1 G185; Bruggemann *et al.*, 1992) and human epidermoid carcinoma (KB3 and KB-V1) cell lines were obtained from Dr M M Gottesman (National Institute of Cancer/National Institute of Health, Bethesda, MD, USA). P388 murine leukaemia, F4–6 Friend murine erythroleukaemia, K562 human erythroleukaemia cells were obtained from Dr A Schaefer (Hamburg University Medical School, Hamburg, Germany). NIH 3T3 cells and KB cell lines were cultured in Dulbecco's modified Eagle medium (DMEM) under standard conditions, while other cells were grown in RPMI media. Culturing media were obtained from Gibco/Life Technologies (Gaithersburg, MD, USA), and supplemented with 10% fetal bovine serum (Sebak, Aidenbach, Germany), 5 mM glutamine, 100 units $ml^{-1}$ penicillin and 100 μg $ml^{-1}$ streptomycin (Sigma Chemical , St. Louis, MO, USA). Drug-selected cell lines were reselected by culturing for 3 days in standard media containing cytostatic agents. P388 and K562 cells were grown in media containing 50 ng $ml^{-1}$ and 100 ng $ml^{-1}$ doxorubicin (Sigma) respectively. KB-V1 cells were cultured in media containing 500 ng $ml^{-1}$ vincristine (Eli Lilly, Indianapolis, IN, USA). In order to achieve different levels of drug resistance the selection procedure was repeated several times.

Recombinant baculovirus carrying the human MDR1 gene was generated, and the Sf9 (*Spodoptera frugiperda*) cells were infected by the MDR1 baculovirus and cultured according to the procedures described previously (Germann *et al.*, 1990).

### *Separation of mononuclear cells of leukaemia patients*

Freshly drawn peripheral blood of leukaemia patients was diluted with an equal volume of HPMI medium (Homolya *et al.*, 1993) and overlaid on Ficoll–Histopaque-1077 (Sigma). The suspension was centrifuged at 1500 *g* for 15 min at room temperature, the mononuclear cell-containing interface was transferred to another tube, diluted with excess HPMI medium, washed twice and finally resuspended in HPMI medium.

### *Quantitative immunoblotting*

Electrophoresis and immunoblotting with the 4077 polyclonal antibody, which recognises both mouse and human MDR1 (Tanaka *et al.*, 1990), were carried out as described in Sarkadi *et al.* (1992). The second antibody was an anti-rabbit, horse radish peroxidase (HRP)-conjugated goat IgG (Jackson Immunoresearch, West Grove, PA, USA), used in 20 000 × dilution. HRP-dependent luminescence on the polyvinyl difluoride (PVDF) membrane immunoblots (ECL supplied by Amersham, Braunschweig, Germany) was determined by excising the respective bands from the PVDF membrane and measuring their luminescence in a liquid scintillation counter (Beckman LS 6000, Single Photon Monitor mode). The amounts of the expressed MDR1 were calculated from the luminescence values, based on a calibration by a dilution series of standard Sf9-MDR1 membrane preparations. By using this method a wide range of luminescence intensities (over three orders of magnitude) could be detected with high accuracy.

### *Fluorometry*

Calcein accumulation was measured by incubating $2.5 \times 10^5$ cells $ml^{-1}$ in HPMI medium containing 0.25 μM calcein AM (Molecular Probes, Eugene, OR, USA). Fluorescence was measured at 37°C with gentle stirring in a Hitachi F-4000 fluorescence spectrophototmeter (excitation and emission wavelengths for calcein were 493 and 515 nm respectively, with a band width of 5 nm). Calibration of dye concentration was based on the measurement of free calcein fluorescence in the same instrument under identical conditions. All experiments were repeated at least three times with each batch of cell preparations.

### *Flow cytometry*

For immunofluorescence staining $8 \times 10^5$ cells were incubated in HPMI medium containing 1% bovine serum albumin (BSA) (Sigma) with the monoclonal antibody, UIC2 (10 μg $ml^{-1}$), which reacts with extracellular epitope(s) of the MDR1 protein (Mechetner and Roninson, 1992). Labelling was performed at 4°C for 45 min, and the cells were then washed twice with HPMI containing 1% BSA, and once with HPMI. Thereafter 17 μg $ml^{-1}$ anti-mouse–FITC antibody conjugate (Dako, Glostrup, Denmark) was applied, similarly to the first antibody. Finally the cells were resuspended in HPMI. Cellular fluorescence was measured with a Cytoronabsolute (Ortho, Ortho Diagnostics Systems, NJ, USA) flow cytometer.

For flow cytometry measurements of calcein uptake, $2 \times 10^5$ cells $ml^{-1}$ were preincubated with 100 μM verapamil or with solvent dimethyl sulphoxide (DMSO) for 5 min at 25°C before the dye loading procedure. Thereafter 0.25 μM

calcein AM (final concentration in the HPMI medium) was added and the cells were incubated for 10 min at 37°C. Non-living cells were detected and gated out by propidium iodide staining. Data were analysed by the Winlist software (Verity Software House, Topsham, ME, USA).

*Drug resistance*

Drug resistance of the various control and MDR1-containing cell types was determined by cell counting after a 72 h incubation of the cells in 24-well plates in the respective culture media, supplemented with various concentrations of the cytostatic agents.

**Figure 1** Quantitative detection of MDR1 expression by Western blotting in various cell lines by the polyclonal antibody, 4077. Increasing levels of P-gp expression have been detected (**a**) in a series of human epidermoid carcinoma cell lines (KB3 non-resistant; KB-V1$_a$ drug-resistant cells; KB-V1$_{b-c}$ generated by further cytostatic drug selections from KB-V1$_a$). (**b**) demonstrates MDR1 expression in NIH 3T3 control cells, MDR1-transfected NIH 3T3 G185 cell line; as well as increasingly drug-resistant murine leukaemia cell line (P388 C: non-resistant, MDR$_a$: less resistant, MDR$_b$: highly resistant). As reference for quantitative estimation of P-gp expression, Sf9 cells expressing human MDR1 were used. Both panels represent luminograms of the peroxidase-stained blots, where each lane contained 20 µg of cellular protein, except the last lane on (**b**), which contained 2 µg of protein of Sf9 cell membranes. The numbers below the lanes represent the determined P-gp amounts in µg MDR1 mg$^{-1}$ cellular protein units. The $IC_{50}$ values obtained for these cell types with doxorubicin in a 3 day growth test were as follows: KB3, <2 nM; KB-V1$_a$, 20 nM; KB-V1$_b$, 60 nM; KB-V1$_c$, 100 nM; NIH 3T3, 5 nM; NIH 3T3 G185, 200 nM; P388C, <1 nM; P388-MDR$_a$, 4 nM; P388-MDR$_b$, 9 nM.

### Results and discussion

The major aim of the present work was to establish a correlation between the expression level of the MDR1 protein and its functional consequence, the resistance to cytotoxic agents in a wide variety of cell types. Therefore we followed in parallel experiments P-gp expression, drug resistance and dye pumping activity in several tumour cell lines. Human epidermoid carcinoma (KB) and erythroleukaemia (K562), as well as murine P388 leukaemia and Friend erythroleukaemia (F4-6) cells were examined. All these cell lines had parent, non-resistant (control) and multidrug-resistant (MDR) counterparts, the latter with a great variety of drug resistance, achieved by continuous drug selection. Since MDR1 expression was variable in these cell lines, all parallel determinations of P-gp expression and function were carried out in samples obtained from the same cell batches. As a reference, we used NIH 3T3 mouse fibroblasts and cells from the same line stably transfected with human MDR1 cDNA via a retroviral vector (Bruggemann *et al.*, 1992). For the quantitative estimation of the MDR1 protein on the immunoblots, we used the insect (Sf9) cell expression system (Germann *et al.*, 1990).

Figure 1a represents Western blot detection of the P-gp in protein extracts of human epidermoid carcinoma (KB) cell lines with increasing levels of drug resistance. Figure 1b shows the immunoblot detection of P-gp in control and MDR1-transfected NIH 3T3 cells, in control and doxorubicin selected P388 mouse leukaemia cells, and in human MDR1-expressing Sf9 cell membranes. In these experiments we used a previously established method to ensure a full electroblotting transfer of the large, heavily glycosylated MDR protein to PVDF membranes (Sarkadi *et al.*, 1992), and applied an anti-human MDR1 polyclonal antibody (4077) that recognises mouse MDR1 but does not show cross-reaction with MDR2 (Tanaka *et al.*, 1990), or, as demonstrated on Figure 1, with any other cellular protein.

**Figure 2** Fluorometric time course of calcein accumulation in drug-sensitive (P388-C) and increasingly drug-resistant (P388-MDR$_{a-b}$) cells. The cells were incubated in the presence of 0.25 µM calcein AM and fluorescence was followed by spectrofluorometry. After 5 min of incubation, a multidrug resistance reversing agent, verapamil (100 µM) was added to the medium. Data of a representative experiment are plotted as fluorescence (in arbitrary units) against time.

The immunoreactive bands in Figure 1 represent the MDR1 protein, its glycosylated form running at an apparent $M_r$ of about 170 kDa, except the underglycosylated form expressed in Sf9 cells, with an $M_r$ of about 130 kDa. Based on the quantitative luminescence measurements and the known amount of MDR1 protein in the isolated Sf9 cell membranes (Sarkadi *et al.*, 1992), MDR1 expression levels in the different cell lines could be determined. The mean values obtained in at least three different measurements for the various cell lines are presented under the respective lanes. In the case of mouse MDR1 antibody detection may be less efficient, thus an underestimation of the amount of MDR1 may occur. However, the validity of these measurements is supported by the finding that another polyclonal anti-human MDR1 antibody (4007, prepared against the C-terminal cytoplasmic domain; Tanaka *et al.*, 1990) provided a similar value for MDR1 expression in all cell types examined (data not shown).

The function of the multidrug transporter in these cell lines was studied by using the calcein assay (Holló *et al.*, 1994), combined with direct cell-survival studies. As previously demonstrated, calcein trapping is slower in the MDR1 expressing cells, owing to the extrusion of calcein AM by the multidrug transporter. When calcein AM extrusion is blocked by an agent that interferes with MDR1 (e.g. verapamil), free calcein rapidly accumulates. As shown in Figure 2 in a representative experiment, the decrease in dye accumulation rate correlates with the level of MDR1 expression. In the control P388 murine leukaemia cells, in the presence of 0.25 μM calcein AM, calcein accumulation is rapid and the addition of 100 μM verapamil has practically no effect either on calcein fluorescence or on its accumulation rate. In contrast, in the MDR1-expressing $P388_a$ cells, calcein accumulation is slower, and verapamil restores the dye uptake rate to that found in control cells, showing that the decreased dye accumulation is due to the MDR1 function. This difference is even larger in the excessively drug-selected $P388_b$ cells, in good correlation with the amount of the expressed MDR1 protein (see Figure 1b).

As shown in Figure 2, the slopes of fluorometric curves are identical after the addition of verapamil, suggesting that the reversal of reduced calcein accumulation was complete and the applied concentration of verapamil did not modify the cellular esterase activity. Although the affinity of calcein AM to P-gp is higher than that of verapamil, a 10-fold excess of the reversing agent was sufficient for complete blocking of calcein AM extrusion (see Homolya *et al.*, 1993). In order to ensure a quantitative evaluation, the highest concentration of verapamil (but still without non-specific effect on the membrane permeability and/or cellular esterase activity) was applied in these and the following experiments and we found that calcein accumulation in the various control cell lines was not affected by verapamil up to a concentration of 150 μM.

**Figure 3** Flow cytometry detection of cell-surface expression of MDR1 (**a**) and calcein accumulation (**b**) in drug sensitive KB3, and increasingly drug resistant $KB\text{-}V1_{a-c}$ cell lines. The cells were labelled (**a**) with a human MDR1-specific monoclonal antibody, UIC2, thereafter a FITC-conjugated anti-mouse second antibody was applied. Cellular green fluorescence intensity was determined by flow cytometry. Representative data are shown as cell numbers plotted against log green fluorescence. Filled histograms show the UIC2 labelled cells, while the isotype controls for each cell line are indicated as outlines. The cells from the same lines were loaded (**b**) with 0.25 μM calcein in the absence ■ or presence □ of 100 μM verapamil. The verapamil-treated cells are shown as an outline on the histogram. Increasing level of MDR1 expression caused decreasing level of calcein accumulation in the absence of verapamil, while verapamil restored dye accumulation up to the control level.

When cell survival was measured in media containing different concentrations of doxorubicin, the $IC_{50}$ values for the above-presented three P388 cell lines were 0.7, 3.7 and 9.0 ng doxorubicin $ml^{-1}$ respectively. These resistance factors were in good correlation with the results obtained from the fluorometric curves shown in Figure 2. A similar close correlation between MDR1 expression, drug resistance and calcein accumulation was observed in several other cell lines (see the legend to Figure 1 and below).

Since the plasma membrane insertion of the MDR1 is required for its drug-extrusion activity, in parallel flow cytometry measurements we have determined the cell-surface expression and the pumping activity of P-gp. For immunofluorescence labelling the UIC2 monoclonal antibody was applied, which reacts exclusively with extracellular epitope(s) of human P-gp (Mechetner and Roninson, 1992). As demonstrated in Figure 3a, the cell surface labelling by UIC2 is in accordance with the immunoblot data (see Figure 1a). The immunofluorescence labelling was compared with the labelling with an $IgG_1$ isotype control, indicated as an outline. In similar experiments the MDR1 cell-surface expression was determined in several cell lines expressing human P-gp (see below), and a linear correlation was found between UIC2 binding and MDR1 levels measured by quantitative immunoblotting ($r = 0.970$, $P = 1.6 \times 10^{-7}$).

Figure 3b demonstrates the flow cytometry measurements of calcein accumulation in the same series of KB cells as shown in Figure 3a. The cells were incubated for 10 min with calcein AM in the absence and presence of verapamil respectively. As demonstrated, calcein accumulation showed a mirror image of the cell-surface labelling by UIC2, that is the more P-gp was expressed, the lower level of calcein fluorescence was detected. Verapamil restored calcein accumulation to the control level. The sensitivity of the assay in most cases remarkably surpassed that of the cell surface labelling, but both measurements reflected the relative doxorubicin resistance in these different MDR1-expressing KB cells (see the legend to Figure 1).

Since the decrease in dye uptake rate correlates both with MDR1 expression and drug resistance, the calcein assay may provide an opportunity for quantitative determination of the MDR1 function. If dye extrusion is entirely blocked by verapamil, the difference between the dye uptake rates after and before verapamil addition respectively, reflects the pumping activity of the multidrug transporter. In order to eliminate the errors arising from differences in esterase activity, cell volume and other fluctuations in the experimental conditions (e.g. cell number, dye concentration, parameters of the fluorescence equipment, etc.), the difference was normalised to an internal standard, provided by the dye uptake rate after the addition of verapamil. Thus, MDR1 activity was expressed by the following dimensionless empirical paramater:

$$\text{MDR1 activity factor}: \ \text{MAF} = (F^* - F)/F^* \qquad (1)$$

where F* and F designate the dye accumulation rates in the presence and absence of an MDR1 inhibitor respectively. We have examined the appropriate conditions for calcein assay in great detail, and found that at pH values in the media between 7.0 and 7.8, at calcein AM concentrations between 0.1 and 1 $\mu$M and at cell numbers between $5 \times 10^4$ $ml^{-1}$ and $2 \times 10^6$ $ml^{-1}$, the value of the MDR1 activity factor remained practically unchanged. Free calcein leakage was found to be extremely slow, its half-time being over 5 h at 25 and 37°C, both in the control and the MDR1-expressing cells. We have also examined the effects of various inhibitors of MDR1 (e.g. vinblastine, vanadate, cyclosporin A) for calcein accumulation (see Holló *et al.*, 1994), and found that whenever maximum inhibition was achieved, the nature of inhibition (competitive or non-competitive) did not influence the obtained MAF value. In addition to the complete blocking of calcein AM extrusion, the important features of the MDR1 inhibitor applied in this test should be, that in the concentrations used:

(i) it should not interfere with the fluorescence of calcein;
(ii) it should not inhibit cellular esterase activity;
(iii) it should not change the non-specific membrane permeability for calcein.

Figure 4a demonstrates in various cell types the correlation between the MAF values calculated from the fluorometric measurements (see Figure 2) and the levels of MDR1 expression determined by quantitative immunoblot-

**Figure 4** Comparison of MDR1 expression and function in various cell types. Control cell lines: P388 C(□), K562 C (▽), KB3 (△), 3T3 C (○); drug-resistant counterparts with different levels of MDR1 expression: P388 $MDR_{a-b}$ (■), K562 $MDR_{a-b}$ (▼), KB-$V1_{a-c}$ (▲), F4-6 (◆), 3T3 MDR1 (●). (**a**) MDR1 expression was quantitatively assessed by Western blotting by using the 4077 polyclonal antibody and the HRP/ECL luminescence method. The MDR1 activity factor (MAF) was calculated for each cell line by using the calcein accumulation rates determined before and after verapamil addition from the fluorometric time courses, as shown in Figure 2, and by using equation (1). The MDR1 activity factor (MAF)±s.d. is plotted against MDR1 contents±s.d. (in $\mu$g MDR1 $mg^{-1}$ cellular protein units). The insert shows the enlarged initial part of the same chart. (**b**) MDR1 expression was assayed by flow cytometry with UIC2 antibody, and MDR1 activity factor (MAF) was determined by flow cytometry calcein assay, as shown in Figure 3. The MAF values were calculated from the absolute fluorescence obtained in the absence and presence of verapamil, and by using equation (1). The MDR1 activity factor (MAF)±s.d. is plotted against UIC2 labelling±s.d. (expressed as the ratio of absolute fluorescence obtained with UIC2 and isotype control immunolabelling respectively). Since MDR1 expression was transient in the drug-selected cell lines, samples for immunodetection and functional assays were obtained from the same cell batches, on the same day. All data points represent at least three independent determinations. For the corresponding $IC_{50}$ values obtained for the different cell types with doxorubicin in a 3 day growth test, see the legend to Figure 1. In the case of the cells previously not shown, the ranges of these $IC_{50}$ values were as follows: K562C, < 1 nM; K562 $MDR_{a-b}$, 5–50 nM; F4-6 MDR, 80–120 nM.

ting (see Figure 1), while Figure 4b shows the MAF values obtained in flow cytometry studies, correlated with the MDR1 cell-surface expression determined by UIC2 labelling (in this case only human MDR1 is detected). For the estimation of the MDR1 activity (MAF) factor from the flow cytometry data, the absolute fluorescence values were calculated from the mean channel values, and inserted into equation (1). Although in this case the MAF parameter was calculated from mean values of 10 min accumulation rates, the results were identical to those obtained from cell population measurements in a spectrofluorimeter.

As shown in these studies, at low MDR1 expression levels the value of MAF (providing an estimate of the pumping activity) is proportional to the level of protein expression, whereas at high MDR1 levels MAF converges to 1 by nature. Thus the sensitivity of this MDR1 activity factor is the highest at lower MDR1 expression levels, correlating with a lower relative resistance to doxorubicin (see the legends to Figures 1 and 4). In all the cell lines examined here it has been previously demonstrated in detail that the expression levels of the MDR1 protein closely correlated with the cellular resistance to a wide variety of various hydrophobic drugs (Bruggemann *et al.*, 1992). Since calcein AM is a high-affinity substrate of the MDR1 transporter (Homolya *et al.*, 1993), the calcein accumulation assay is suggested to provide an overall reflection of the cellular multidrug resistance.

The data presented here clearly demonstrate that the level of P-gp expression in most cases shows a good correlation with its function. However, the occurrence of a non-functional P-gp may be misleading for conclusions related to multidrug resistance in a given cell type, and only a functional test may help in obtaining such a clinically relevant laboratory diagnosis. In fact, in a K562 cell line (generated in our laboratory), the expression of a non-functional mutant of P-gp can be detected by immunoblotting and UIC2 flow cytometry, but does not exhibit calcein AM extrusion (data not shown). Another possibility, leading to a positive calcein accumulation-derived MAF value without MDR1 expression is the appearance of MRP (multidrug-resistance associated protein; Cole *et al.*, 1992), which has recently been reported to extrude calcein from tumour cells (Feller *et al.*, 1995*a b*; Twentyman, personal communication). In this latter case the specific antibody labelling and the selective inhibition of the MDR- and/or MRP-dependent calcein transport should help the proper diagnosis (see Feller *et al.*, 1995*b*).

The major goal of the above-described work has been to introduce a quantitative assay panel for the rapid and simple *in vitro* assessment of multidrug resistance in cells obtained from patients suffering from cancer. Representative measurements with lymphocytes of a patient with chronic lymphocytic leukaemia are shown in Figure 5. The cell-surface expression of P-gp was detected by UIC2 (Figure 5a), indicating a 1.15 times increase in labelling, as compared with the isotype control. In a parallel experiment the calcein accumulation assay in the same sample (Figure 5b) showed a clear measurable difference in the absence and presence of verapamil respectively, yielding a MAF of 0.31. According to our preliminary data obtained with leucocyte samples of haematological patients, the clinically drug-resistant cells show relatively low MDR1 expression, and are in the MAF=0.05–0.5 range. As indicated by Figure 4, this is the range where the MAF is proportional to P-gp expression and the sensitivity of the method is the highest.

The above detailed analysis of multidrug resistance in various cell lines suggests that a combination of cell-surface MDR1 expression analysis, e.g. with monoclonal antibody UIC2, and a quantitative functional test, e.g. by the calcein accumulation assay with the calculation of MAF described here, provides a safe way of quantitatively assessing the level of multidrug resistance in clinical samples. In addition, the favourable optical properties of calcein allow dual fluorescence studies to be performed, in which the functional assay is carried out in surface marker-selected leukaemic (or other tumour) cells. The unique restriction for such studies is that the excitation and/or emission wavelengths of the fluorophore of the tumour marker has to be remarkably dissimilar to that of calcein (FITC and R-phycoerythrine conjugates are incompatible). A detailed clinical study of multidrug resistance in isolated leucocytes of haematological patients, by using the above described assays, is underway in our Institute.

**Figure 5** UIC2/FITC labelling and calcein accumulation properties of leucocytes of a patient suffering from chronic lymphocytic leukaemia. Ficoll-separated leucocytes were labelled by the UIC2 monoclonal antibody, while another portion of the sample was examined by the calcein accumulation assay. The FITC surface labelling and the cellular calcein fluorescence were detected by flow cytometry. (**a**) UIC2/FITC antibody labelling of the leukaemic cells of a chronic lymphocytic leukaemia (CLL) patient (dark grey histogram); the light-grey histogram shows the labelling by the isotype control/FITC antibodies. Histograms are depicted as an overlay. (**b**) The cells from the same clinical sample were loaded with 0.25 μM calcein in the absence or presence of 100 μM verapamil. The verapamil-pretreated cells are shown in dark grey, whereas the cellular fluorescence of the cells pretreated only with solvent, is shown by the light-grey histogram.

**Abbreviations**

AM acetoxymethyl ester; BSA, bovine serum albumin; CLL, chronic lymphocytic leukaemia; FCS, fetal calf serum; FITC, fluorescein-5-isothiocyanate; HRP, horseradish peroxidase; $IC_{50}$, concentration causing 50% inhibition; MDR1, multidrug resistance protein; MAF, multidrug resistance activity factor; MRP, multidrug resistance-associated protein; PBS, phosphate-buffered saline; PVDF, polyvinylidene difluoride; TCA, trichloroacetic acid.

**Acknowledgements**
We thank Dr G Gárdos (NIHBT, Budapest) and Dr R C Boucher (UNC, Chapel Hill) for their valuable advice; and R Mihalik and Dr M Benczúr for their help in flow cytometry measurements. We are grateful to M M Gottesman (NCI, NIH) for providing the NIH 3T3 MDR1 G185 cell line and to A Schaefer for providing several drug-selected cell lines. The technical help of Andrea Siposs and Ilona Zombori is gratefully acknowledged. This work was supported by research grants from OTKA F 13178, ACCORD and OMFB (Mecenatura), Hungary.

### References

ALTENBERG GA, VANOYE CG, HORTON JK AND REUSS L. (1994). Undirectional fluxes of rhodamine 123 in multidrug-resistant cells: Evidence against direct drug extrusion from the plasma membrane. *Proc. Natl Acad. Sci. USA*, **91**, 4654–4657.

BORNMANN WG AND ROEPE DR. (1994). Analysis of drug transport kinetics in multidrug-resistant cells using a novel coumarin–vinblastine compound. *Biochemistry*, **33**, 12665–12675.

BRUGGEMANN EP, CURRIER SJ, GOTTESMAN MM AND PASTAN I. (1992). Characterization of the azidopine and vinblastine binding site of P-glycoprotein. *J. Biol. Chem.*, **267**, 21020–21026.

COLE SPC, BHARDWAY G, GERLACH JH, MACKIE JE, GRANT CE, ALMQUIST KC, STEWART AJ, KURTZ EU, DUNCAN AMV AND DEELEY GG. (1992). Overexpression of a novel transporter gene in a multidrug resistant human lung cancer cell line. *Science*, **258**, 1650–1654.

CHAUDHARY PM AND RONINSON IB. (1991). Expression and activity of P-glycoprotein, a multidrug efflux pump, in human hematopoietic stem cells. *Cell*, **66**, 85–94.

FELLER N, BROXTERMAN HJ, WÄHRER DCR AND PINEDO HM. (1995*a*). ATP-dependent efflux of calcein by the multidrug resistance protein (MRP): no inhibition by intracellular glutathione depletion. *FEBS Lett.*, **368**, 385–388.

FELLER N, KUIPER CM, LANKELMA J, RUHDAL JK, SCHEPER RJ, PINEDO HM AND BROXTERMAN HJ. (1995*b*). Functional detection of MDR1/P170 and MRP/P190-mediated multidrug resistance in tumour cells by flow cytometry. *Br. J. Cancer*, **72**, 543–549.

FREY T, YUE S, HAUGHLAND RP. (1995). Dyes providing increased sensitivity in flow-cytometric dye-efflux assays for multidrug resistance. *Cytometry*, **20**, 218–227.

GERMANN UA, WILLINGHAM MC, PASTAN I AND GOTTESMAN MM. (1990). Expression of the human multidrug transporter in insect cells by a recombinant baculovirus. *Biochemistry*, **29**, 2295–2303.

GOLDSTEIN LJ, PASTAN I AND GOTTESMAN MM. (1992). Multidrug resistance in human cancer. *Crit. Rev. Oncol. Haematol.*, **12**, 243–253.

GOTTESMAN MM AND PASTAN I. (1993). Biochemistry of multidrug resistance mediated by the multidrug transporter. *Annu. Rev. Biochem.*, **62**, 385–427.

HAUGHLAND RP AND LARISON KD (EDS). (1992). Low molecular weight tracers: fluorescent dyes for assessing vital cell functions. In *Handbook of Fluorescent Probes and Research Chemicals 1992–1994*, pp. 163, 172–173. Molecular Probes: Eugene, OR.

HERWEIJER H, ENGH G AND NOOTER K. (1989). A rapid and sensitive flow cytometric P-glycoprotein method for the detection of multi-drug resistant cells. *Cytometry*, **10**, 463–468.

HOLLÓ ZS, HOMOLYA L, DAVIS CW AND SARKADI B. (1994). Calcein accumulation as a fluorometric functional assay of the multidrug transporter. *Biochim. Biophys. Acta*, **1191**, 384–388.

HOMOLYA L, HOLLÓ ZS, GERMANN UA, PASTAN I, GOTTESMAN MM AND SARKADI B. (1993). Fluorescent cellular indicators are extruded by the multidrug resistance protein. *J. Biol. Chem.*, **268**, 21493–21496.

LEE JS, PAULL K, ALVAREZ M, HOSE C, MONKS A, GREVER M, FOJO AT AND BATES SE. (1994). Rhodamine efflux patterns predict P-glycoprotein substrates in the National Cancer Institute Drug Screen. *Mol. Pharmacol.*, **46**, 627–638.

LELONG IH, GUZIKOWSKI AP, HAUGLAND RP, PASTAN I, GOTTESMAN MM AND WILLINGHAM MC. (1991). Fluorescent verapamil derivative for monitoring activity of the multidrug transporter. *Mol. Pharmacol.*, **40**, 490–494.

MECHETNER EB AND RONINSON IB. (1992). Efficient inhibition of P-glycoprotein mediated multidrug resistance with a monoclonal antibody. *Proc. Natl Acad. Sci. USA*, **89**, 5824–5828.

NEYFAKH AA. (1988). Use of fluorescent dyes as molecular probes for the study of multidrug resistance. *Exp. Cell Res.*, **174**, 168–174.

ROEPE PD. (1992). Analysis of the steady state and initial rate of doxorubicin efflux from a series of multidrug-resistant cells expressing different levels of P-glycoprotein. *Biochemistry*, **31**, 12555–12564.

ROEPE PD, WEI LY, CRUZ J AND CARLSON D. (1993). Lower electrical membrane potential and altered $pH_i$ homeostasis in multidrug-resistant (MDR) cells: further characterization of a series of MDR cell lines expressing different levels of P-glycoprotein. *Biochemistry*, **32**, 11042–11056.

SARKADI B, PRICE EM, BOUCHER RC, GERMANN UA AND SCARBOROUGH GA. (1992). Expression of the human multidrug resistance cDNA in insect cells generates a high activity drug-stimulated membrane ATPase. *J. Biol. Chem.*, **267**, 4854–4858.

SPOLESTRA EC, WESTERHOFF HV, DEKKER H AND LANKELMA J. (1992). Kinetics of daunorubicin transport by P-glycoprotein of intact cells. *Eur. J. Biochem.*, **207**, 567–579.

TANAKA S, CURRIER SJ, BRUGGEMANN EP, GERMANN UA, PASTAN I AND GOTTESMAN MM. (1990). Use of recombinant P-glycoprotein fragments to produce antibodies to the multidrug transporter. *Biochem. Biophys. Res. Comm.*, **166**, 180–186.

THIEBAUT F, CURRIER SJ, WHITAKER J, HAUGHLAND RP, GOTTESMAN MM, PASTAN I AND WILLINGHAM MC. (1990). Activity of the multidrug transporter results in alkalinization of the cytosol: measurement of cytosolic pH by microinjection of a pH-sensitive dye. *J. Histochem. Cytochem.*, **38**, 685–690.

WALL DM, HU XF, ZALCBERG JR AND PARKIN JD. (1991). Rapid functional assay for multidrug resistance in human tumor cell lines using the fluorescent indicator Fluo-3. *J. Natl Cancer Inst.*, **83**, 206–207.

WALL DM, SPARROW R, HU XF, NADALIN G, ZALCBERG JR, MARSCHNER IC, VAN DER WEYDEN M AND PARKIN JD. (1993). Clinical application of a rapid, functional assay for multidrug resistance based on accumulation of the fluorescent dye, Fluo-3. *Eur. J. Cancer*, **29**, 1024–1027.

WEAVER JL, PINE PS, ASZALOS A, SCHOENLEIN PV, CURRIER SJ, PADMANABHAN R AND GOTTESMAN MM. (1991). Laser scanning and confocal microscopy of daunorubicin, doxorubicin, and rhodamine 123 in multidrug-resistant cells. *Exp. Cell Res.*, **196**, 323–329.